# 2-[4-(2-Chloro­benz­yl)-3-methyl-6-oxo-1,6-di­hydro­pyridazin-1-yl]-*N*-(4-fluoro­phen­yl)acetamide

**DOI:** 10.1107/S241431462300901X

**Published:** 2023-10-19

**Authors:** Hamza Assila, Issam Ameziane El Hassani, Abderrazzak El Moutaouakil Ala Allah, Abdulsalam Alsubari, Joel T Mague, Youssef Ramli, MHammed Ansar

**Affiliations:** a Laboratory of Medicinal Chemistry, Drug Sciences Research Center, Faculty of Medicine and Pharmacy, Mohammed V University in Rabat, Morocco; b Laboratory of Medicinal Chemistry, Faculty of Clinical Pharmacy, 21 September University, Yemen; c Department of Chemistry, Tulane University, New Orleans, LA 70118, USA; d Mohammed VI Center for Research and Innovation (CM6), Rabat 10000, Morocco; University of Aberdeen, United Kingdom

**Keywords:** crystal structure, pyridazine, hydrogen bond, π-stacking, aryl­acetamide, crystal structure

## Abstract

The conformation of the title mol­ecule, C_20_H_17_ClFN_3_O_2_, is partly determined by an intra­molecular C—H⋯O hydrogen bond, which leads to a dihedral angle of 14.7 (4)° between the fluoro­benzene ring and the acetamide group. The 2-chloro­benzyl group is rotationally disordered over two orientations in a 0.656 (2): 0.344 (2) ratio. In the crystal, a layered structure is formed by N—H⋯O, C—H⋯O and C—H⋯F hydrogen bonds plus slipped π–π stacking inter­actions.

## Abstract 2

An intra­molecular C—H⋯O hydrogen bond helps to determine the conformation of the title mol­ecule in which the 2-chloro­benzyl group is rotationally disordered.

## Graphical abstract

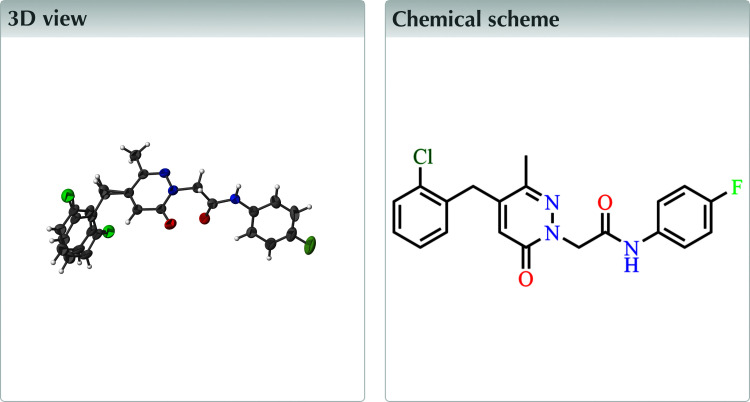

## Structure description

Pyridazinone derivatives possess a number of biological activities including anti-oxidant (Khokra *et al.*, 2016 ▸), anti-bacterial and anti­fungal (Abiha *et al.* 2018 ▸), anti-cancer (Kamble *et al.* 2017 ▸), analgesic and anti-inflammatory (Ibrahim *et al.* 2017 ▸), anti-depressant (Boukharsa *et al.* 2016 ▸) and anti-ulcer properties (Yamada *et al.*, 1981 ▸). In addition, *N*-aryl­acetamide derivatives with their wide spectrum of activities (*e.g.*, Missioui *et al.*, 2022 ▸) have significant importance as inter­mediates in organic chemistry. As a continuation of a our work in synthesizing new *N*-aryl­acetamide derivatives (*e.g.*, Mortada *et al.*, 2023 ▸), and developing new pyridazine-3(2*H*)-one compounds (*e.g.*, Zaoui *et al.*, 2022 ▸), the title compound C_20_H_17_ClFN_3_O_2_ was synthesized and its crystal structure is reported here.

**Figure 1:**
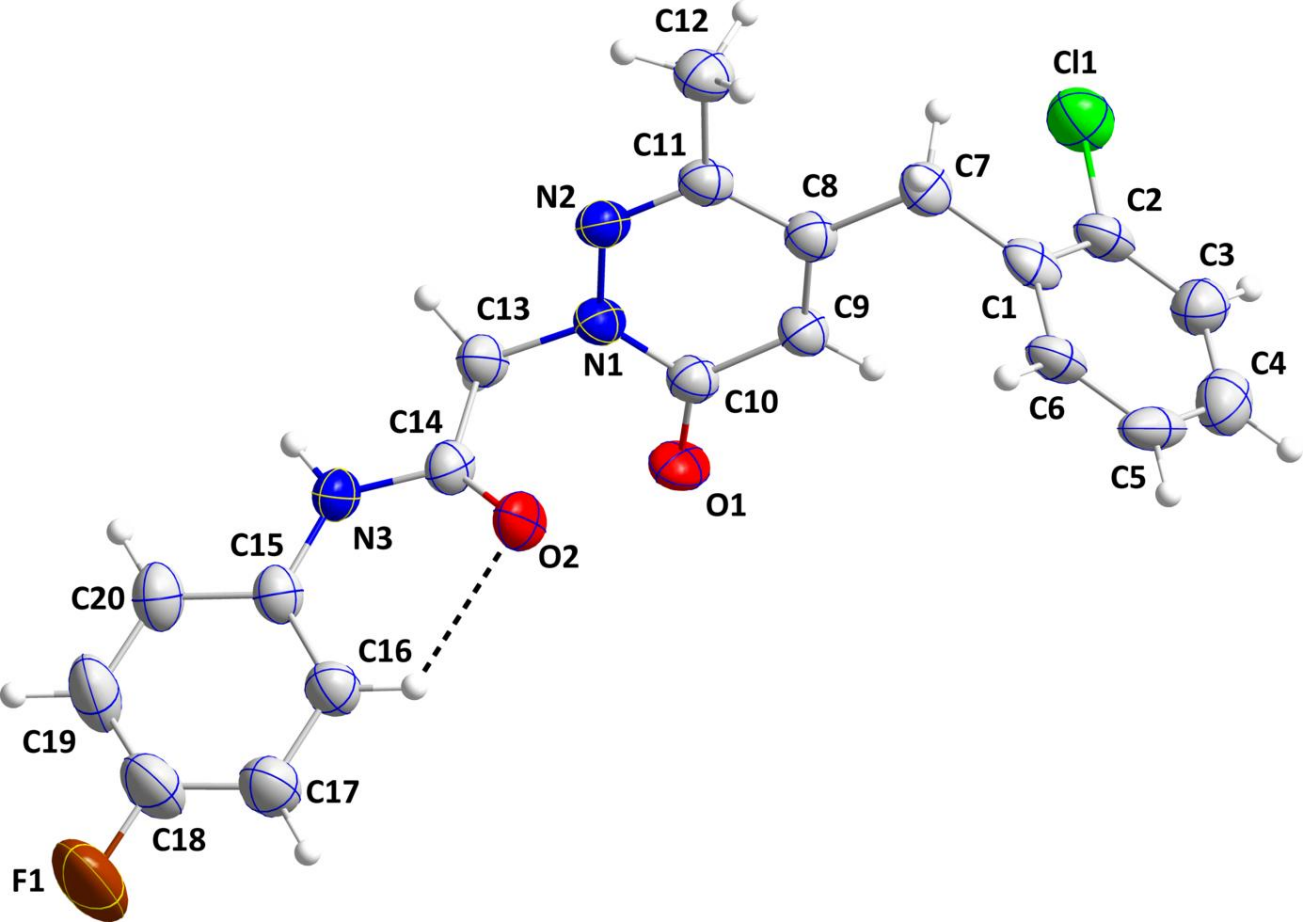
The title mol­ecule with labeling scheme and 50% probability ellipsoids. The intra­molecular C—H⋯O hydrogen bond is depicted by a dashed line and only the major component of the disorder is shown.

The title mol­ecule adopts an ‘extended’ conformation with a dihedral angle between the mean plane of the C15–C20 fluoro­benzene ring and that defined by N2, C14, C13 and O2 of 14.7 (4)°. This is likely due in part to the intra­molecular C16—H16⋯O2 hydrogen bond (Table 1 ▸ and Fig. 1 ▸). The dihedral angle between this latter plane and the mean plane of the C8–C11/N1/N2 ring is 72.07 (16)° while that between the mean planes of the C8–C11/N1/N2 and the C1–C6 rings is 80.38 (16)°. The disorder in this part of the mol­ecule features a 177.2 (5)° rotation of the 2-chloro­phenyl between the two components of the disorder in a 0.656 (2): 0.344 (2) ratio.

**Figure 2:**
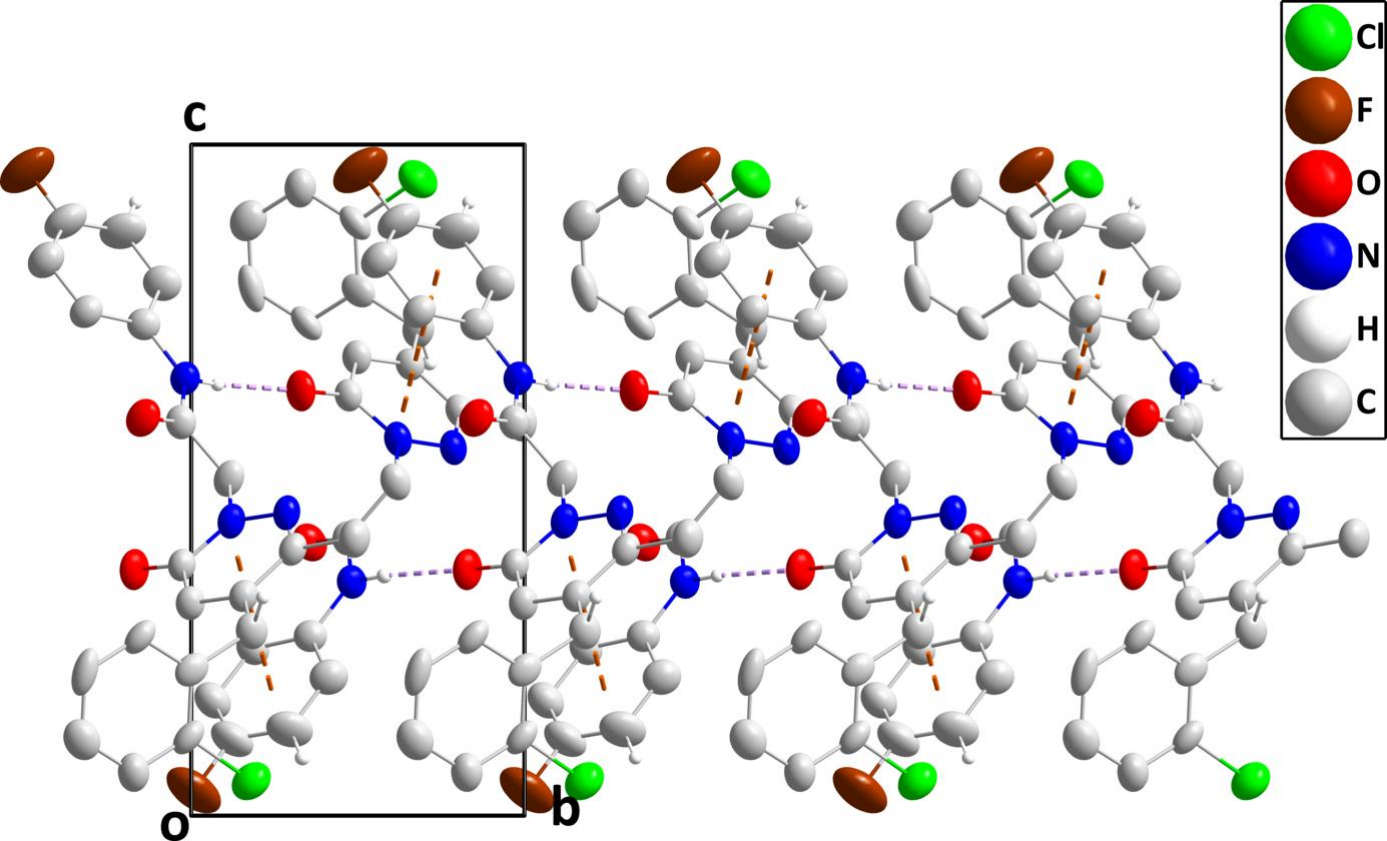
A portion of one chain of mol­ecules viewed along the *a*-axis direction with N—H⋯O hydrogen bonds and slipped, π-stacking inter­actions depicted, respectively, by violet and orange dashed lines. Non-inter­acting hydrogen atoms are omitted for clarity.

**Figure 3:**
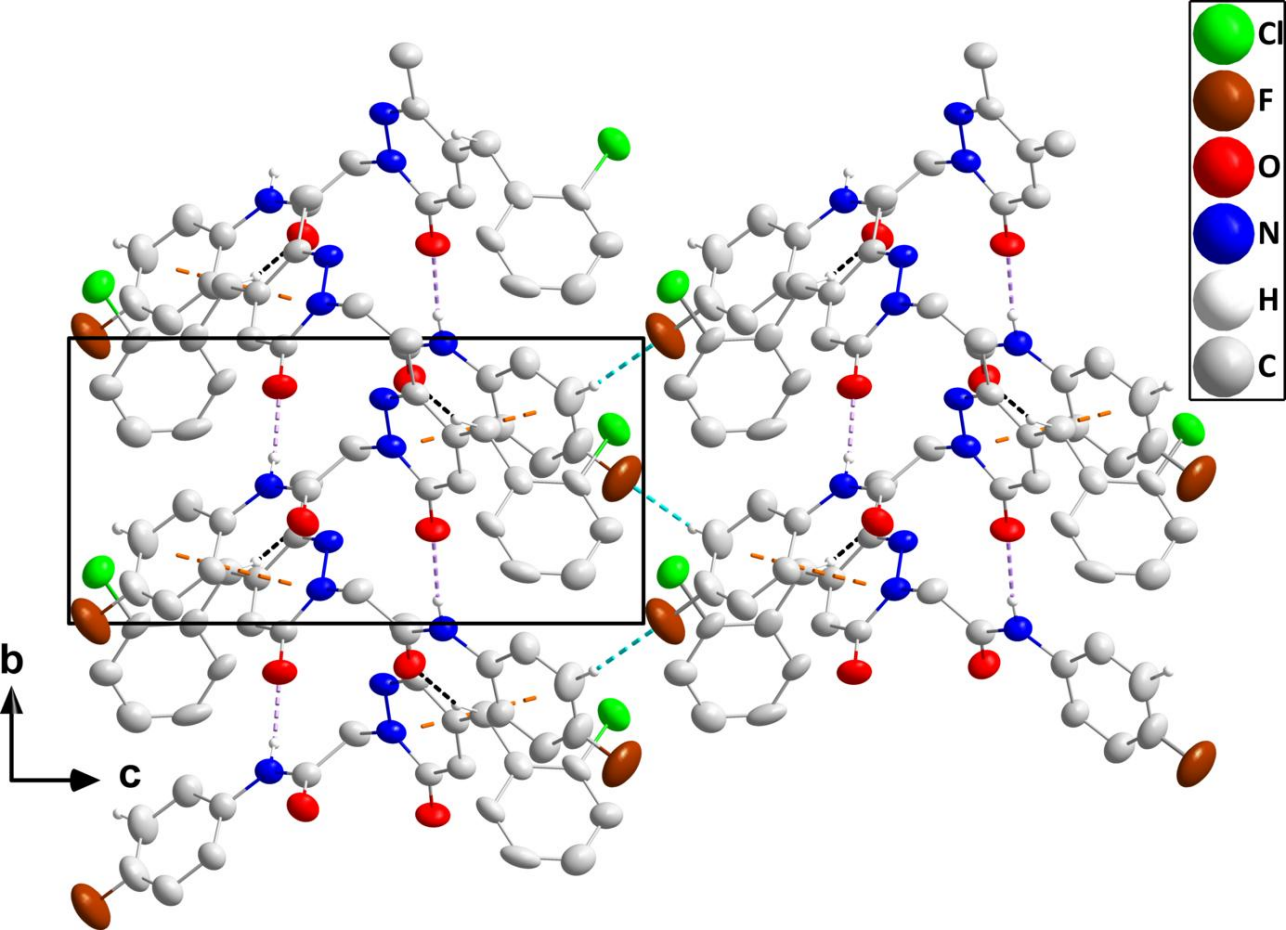
Packing viewed along the *a*-axis direction with N—H⋯O, C—H⋯O and C—H⋯F hydrogen bonds depicted, respectively, by violet, black and light blue dashed lines. Slipped π-stacking inter­actions are depicted by orange dashed lines and non-inter­acting hydrogen atoms are omitted for clarity.

In the crystal, N3—H3⋯O1 hydrogen bonds form chains of mol­ecules extending along the *b*-axis direction. These are reinforced by slipped π-stacking inter­actions between a pyridazine and a 4-fluoro­phenyl ring at –*x* + 2, *y* + 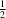 , –*z* + 1 [centroid–centroid separation = 3.706 (3) Å, dihedral angle = 8.7 (2)°, slippage = 1.18 Å] (Fig. 2 ▸). These chains are connected into layers by C19—H19⋯F1 hydrogen bonds with the layers further connected by C7—H7*A*⋯O2 hydrogen bonds (Fig. 3 ▸).

## Synthesis and crystallization

A mixture of 3-benzyl­idene-4-oxo­penta­noic acid derivative (0.010 mol) and hydrazine hydrate (0.020 mol) in ethanol was refluxed to obtain the 5-(2-chloro­benz­yl)-6-methyl­pyridazin-3(2*H*)-one precursor. To this pyridazine-3(2*H*)-one (0.010 mol) was added 0.010 mol of 2-chloro-*N*-(4-fluoro­phen­yl)acetamide, followed by 0.020 mol of potassium bicarbonate and a spatula tip of BTBA (benzyl­tri­butyl­ammonium bromide). The mixture was kept stirring at room temperature for 24 h and the progress of the reaction was monitored by TLC. Then, 200 ml of distilled water were added to the reaction mixture, the precipitated product was filtered off, dried and recrystallized from acetone solution to yield colorless crystals of the title compound.

Yield 82%; m.p: 477–479 K. ^1^H NMR [300 MHz DMSO-*d*_6_, δ(p.p.m.)]: 2.25 (*s*, 3H, CH_3_); 3.96 (*s*, 2H, phenyl-CH_2_-pyridazinone); 4,78 (*s*, 2H, N—CH_2_—CO); 6.06 (*s*, 1H, pyridazinone); 7.07–7.58 (*m*, 8H, two phen­yl); 10.32 (*s*, 1H, NH). ^13^C NMR [126 MHz DMSO-*d*_6_, δ(p.p.m.)]: 19.06 (CH_3_); 35.32 (phenyle-CH_2_-pyridazinone); 54.61 (pyridazinone-CH_2_—CO); 115.84 (*d, J* = 22.5 Hz) (C aromatic acetamide); 121.32 (*d, J* = 7.7 Hz) (C aromatic acetamide); 126.55 (CH pyridazinone); 128.32 (C aromatic); 129.73 (C aromatic); 130.17 (C aromatic); 132.08 (C aromatic); 134.02 (C aromatic); 134.87 (C aromatic-CH_2_); 135.67 (*d, J* = 2,5 Hz) (C aromatic-NH); 144.88 (CH_2_—C pyridazinone); 145.96 (C pyridazinone–CH_3_); 157.63 (*d, J* = 293,9 Hz) (C aromatic-F); 159.83 (C pyridazinone=O); 165.68 (NH—C=O). MS (ESI+): *m*/*z* = 386.10.

## Refinement

Crystal data, data collection and refinement details are presented in Table 2 ▸. The *o-*chloro­benzyl group is rotationally disordered over two orientations in a 0.656 (2): 0.344 (2) ratio with the components refined with restraints to make their geometries comparable. One reflection affected by the beamstop was omitted from the final refinement.

## Supplementary Material

Crystal structure: contains datablock(s) global, I. DOI: 10.1107/S241431462300901X/hb4454sup1.cif

Structure factors: contains datablock(s) I. DOI: 10.1107/S241431462300901X/hb4454Isup2.hkl

Supporting information file. DOI: 10.1107/S241431462300901X/hb4454Isup3.cml

CCDC reference: 2301289

Additional supporting information:  crystallographic information; 3D view; checkCIF report

## Figures and Tables

**Table 1:** Hydrogen-bond geometry (Å, °)

*D*—H⋯*A*	*D*—H	H⋯*A*	*D*⋯*A*	*D*—H⋯*A*
N3—H3⋯O1^i^	0.88	1.95	2.815 (5)	168
C7—H7*A*⋯O2^ii^	0.99	2.32	3.282 (15)	164
C16—H16⋯O2	0.95	2.29	2.900 (6)	121
C19—H19⋯F1^iii^	0.95	2.42	3.336 (7)	162

Symmetry codes: (i) 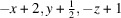 ; (ii) 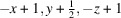 ; (iii) 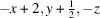 .

**Table 2:** Experimental details

Crystal data	
Chemical formula	C_20_H_17_ClFN_3_O_2_
*M*_r_	385.82
Crystal system, space group	Monoclinic, *P*2_1_
Temperature (K)	150
*a*, *b*, *c* (Å)	10.0602 (13), 6.7704 (9), 14.524 (2)
β (°)	110.168 (2)
*V* (Å^3^)	928.6 (2)
*Z*	2
Radiation type	Mo *K*α
μ (mm^−1^)	0.24
Crystal size (mm)	0.19 × 0.14 × 0.08

Data collection	
Diffractometer	Bruker D8 QUEST PHOTON 3 diffractometer
Absorption correction	Numerical (*SADABS*; Krause *et al.*, 2015 ▸)
*T*_min_, *T*_max_	0.80, 0.98
No. of measured, independent and observed [*I* > 2σ(*I*)] reflections	6266, 2856, 2376
*R*_int_	0.039
θ_max_ (°)	23.9
(sin θ/λ)_max_ (Å^−1^)	0.570

Refinement	
*R*[*F* ^2^ > 2σ(*F* ^2^)], *wR*(*F* ^2^), *S*	0.048, 0.131, 1.03
No. of reflections	2856
No. of parameters	240
No. of restraints	17
H-atom treatment	H-atom parameters constrained
Δρ_max_, Δρ_min_ (e Å^−3^)	0.27, −0.28
Absolute structure	Flack *x* determined using 904 quotients [(*I* ^+^)−(*I* ^−^)]/[(*I* ^+^)+(*I* ^−^)] (Parsons *et al.*, 2013 ▸)
Absolute structure parameter	−0.01 (7)

Computer programs: *APEX4* and *SAINT* (Bruker, 2021 ▸), *SHELXT* (Sheldrick, 2015*a* ▸), *SHELXL2018/1* (Sheldrick, 2015*b* ▸), *DIAMOND* (Brandenburg & Putz, 2012 ▸) and *SHELXTL* (Sheldrick, 2008 ▸).

## full crystallographic data

### Crystal data

**Table:**

C_20_H_17_ClFN_3_O_2_	*F*(000) = 400
*M**_r_* = 385.82	*D*_x_ = 1.380 Mg m^−^^3^
Monoclinic, *P*2_1_	Mo *K*α radiation, λ = 0.71073 Å
*a* = 10.0602 (13) Å	Cell parameters from 3275 reflections
*b* = 6.7704 (9) Å	θ = 3.0–23.6°
*c* = 14.524 (2) Å	µ = 0.24 mm^−^^1^
β = 110.168 (2)°	*T* = 150 K
*V* = 928.6 (2) Å^3^	Plate, colourless
*Z* = 2	0.19 × 0.14 × 0.08 mm

### Data collection

**Table:**

Bruker D8 QUEST PHOTON 3 diffractometer	2856 independent reflections
Radiation source: fine-focus sealed tube	2376 reflections with *I* > 2σ(*I*)
Graphite monochromator	*R*_int_ = 0.039
Detector resolution: 7.3910 pixels mm^-1^	θ_max_ = 23.9°, θ_min_ = 3.0°
φ and ω scans	*h* = −11→11
Absorption correction: numerical (*SADABS*; Krause *et al.*, 2015)	*k* = −7→7
*T*_min_ = 0.80, *T*_max_ = 0.98	*l* = −16→16
6266 measured reflections	

### Refinement

**Table:**

Refinement on *F*^2^	Secondary atom site location: difference Fourier map
Least-squares matrix: full	Hydrogen site location: inferred from neighbouring sites
*R*[*F*^2^ > 2σ(*F*^2^)] = 0.048	H-atom parameters constrained
*wR*(*F*^2^) = 0.131	*w* = 1/[σ^2^(*F*_o_^2^) + (0.0712*P*)^2^ + 0.1965*P*] where *P* = (*F*_o_^2^ + 2*F*_c_^2^)/3
*S* = 1.03	(Δ/σ)_max_ < 0.001
2856 reflections	Δρ_max_ = 0.27 e Å^−^^3^
240 parameters	Δρ_min_ = −0.28 e Å^−^^3^
17 restraints	Absolute structure: Flack *x* determined using 904 quotients [(*I*^+^)-(*I*^-^)]/[(*I*^+^)+(*I*^-^)] (Parsons *et al.*, 2013)
Primary atom site location: dual	Absolute structure parameter: −0.01 (7)

### Special details

**Table:**

Experimental. The diffraction data were collected in three sets of 363 frames (0.5° width in ω) at φ = 0, 120 and 240°. A scan time of 40 sec/frame was used.
Geometry. All esds (except the esd in the dihedral angle between two l.s. planes) are estimated using the full covariance matrix. The cell esds are taken into account individually in the estimation of esds in distances, angles and torsion angles; correlations between esds in cell parameters are only used when they are defined by crystal symmetry. An approximate (isotropic) treatment of cell esds is used for estimating esds involving l.s. planes.
Refinement. Refinement of F^2^ against ALL reflections. The weighted R-factor wR and goodness of fit S are based on F^2^, conventional R-factors R are based on F, with F set to zero for negative F^2^. The threshold expression of F^2^ > 2sigma(F^2^) is used only for calculating R-factors(gt) etc. and is not relevant to the choice of reflections for refinement. R-factors based on F^2^ are statistically about twice as large as those based on F, and R- factors based on ALL data will be even larger. H-atoms attached to carbon were placed in calculated positions (C—H = 0.95 - 0.99 Å) and were included as riding contributions with isotropic displacement parameters 1.2 - 1.5 times those of the attached atoms. That attached to nitrogen was located in a difference map and refined with a DFIX 0.91 0.01 instruction. The *o*-chlorobenzyl group is rotationally disoordered over two sites 177.2 (5)° apart in a 0.656 (2)/0.344 (2) ratio. The two components were refined as rigid hexagons with additional restraints to make their geometries comparable. One reflection affected by the beam stop was omitted from the final refinement.

### Fractional atomic coordinates and isotropic or equivalent isotropic displacement parameters (Å^2^)

**Table:**

	*x*	*y*	*z*	*U*_iso_*/*U*_eq_	Occ. (<1)
Cl1	0.7132 (2)	0.6802 (4)	0.94862 (15)	0.0618 (6)	0.656 (2)
Cl1A	0.3896 (4)	0.3635 (7)	0.6576 (3)	0.0618 (6)	0.344 (2)
F1	0.9061 (5)	0.0075 (7)	0.0379 (3)	0.1107 (17)	
O1	0.9168 (3)	0.3291 (5)	0.6332 (3)	0.0492 (9)	
O2	0.7561 (3)	0.3559 (5)	0.4072 (2)	0.0485 (9)	
N1	0.8281 (4)	0.6205 (5)	0.5611 (3)	0.0390 (9)	
N2	0.7474 (4)	0.7866 (6)	0.5485 (3)	0.0400 (9)	
N3	0.9225 (4)	0.4816 (6)	0.3484 (3)	0.0391 (9)	
H3	0.983416	0.579248	0.357540	0.047*	
C7	0.5560 (15)	0.6816 (12)	0.7246 (10)	0.046 (3)	0.656 (2)
H7A	0.463053	0.714697	0.674956	0.055*	0.656 (2)
H7B	0.585469	0.795035	0.770111	0.055*	0.656 (2)
C1	0.5373 (5)	0.5015 (6)	0.7825 (3)	0.041 (2)	0.656 (2)
C2	0.6067 (5)	0.4920 (7)	0.8834 (3)	0.0432 (13)	0.656 (2)
C3	0.5904 (6)	0.3270 (8)	0.9354 (3)	0.052 (2)	0.656 (2)
H3A	0.637871	0.320515	1.004371	0.063*	0.656 (2)
C4	0.5046 (6)	0.1717 (7)	0.8867 (4)	0.059 (2)	0.656 (2)
H4	0.493389	0.058937	0.922286	0.071*	0.656 (2)
C5	0.4351 (5)	0.1812 (7)	0.7858 (4)	0.056 (2)	0.656 (2)
H5	0.376434	0.075013	0.752518	0.067*	0.656 (2)
C6	0.4514 (5)	0.3461 (8)	0.7338 (3)	0.0432 (13)	0.656 (2)
H6	0.403960	0.352669	0.664833	0.052*	0.656 (2)
C7A	0.583 (4)	0.712 (2)	0.741 (2)	0.046 (3)	0.344 (2)
H7C	0.484915	0.750414	0.700522	0.055*	0.344 (2)
H7D	0.628844	0.828556	0.780181	0.055*	0.344 (2)
C1A	0.5776 (10)	0.5441 (14)	0.8094 (7)	0.041 (2)	0.344 (2)
C2A	0.6600 (10)	0.5581 (14)	0.9082 (7)	0.0432 (13)	0.344 (2)
H2A	0.718157	0.670747	0.931670	0.052*	0.344 (2)
C3A	0.6572 (11)	0.4075 (16)	0.9725 (6)	0.052 (2)	0.344 (2)
H3B	0.713505	0.417063	1.039937	0.063*	0.344 (2)
C4A	0.5721 (13)	0.2427 (14)	0.9381 (8)	0.059 (2)	0.344 (2)
H4A	0.570209	0.139715	0.982019	0.071*	0.344 (2)
C5A	0.4897 (12)	0.2286 (14)	0.8393 (9)	0.056 (2)	0.344 (2)
H5A	0.431564	0.116050	0.815834	0.067*	0.344 (2)
C6A	0.4925 (10)	0.3793 (18)	0.7750 (6)	0.0432 (13)	0.344 (2)
C8	0.6632 (4)	0.6569 (7)	0.6726 (3)	0.0385 (11)	
C9	0.7456 (5)	0.4970 (7)	0.6832 (4)	0.0393 (10)	
H9	0.743747	0.398076	0.729138	0.047*	
C10	0.8370 (4)	0.4717 (7)	0.6265 (3)	0.0374 (10)	
C11	0.6674 (4)	0.8051 (7)	0.6030 (3)	0.0381 (11)	
C12	0.5826 (5)	0.9905 (8)	0.5867 (4)	0.0513 (13)	
H12A	0.606286	1.063382	0.648541	0.077*	
H12B	0.481472	0.957689	0.562809	0.077*	
H12C	0.604076	1.072240	0.537915	0.077*	
C13	0.9116 (5)	0.6132 (7)	0.4974 (4)	0.0429 (12)	
H13A	1.009880	0.574945	0.536509	0.052*	
H13B	0.914605	0.746647	0.470293	0.052*	
C14	0.8535 (5)	0.4690 (7)	0.4135 (3)	0.0393 (11)	
C15	0.9084 (4)	0.3573 (7)	0.2676 (3)	0.0410 (11)	
C16	0.8316 (5)	0.1846 (8)	0.2489 (3)	0.0450 (11)	
H16	0.778599	0.147835	0.289062	0.054*	
C17	0.8311 (6)	0.0641 (9)	0.1718 (4)	0.0572 (14)	
H17	0.779684	−0.056479	0.159180	0.069*	
C18	0.9065 (6)	0.1228 (10)	0.1143 (4)	0.0662 (17)	
C19	0.9804 (7)	0.2951 (11)	0.1287 (4)	0.0687 (17)	
H19	1.029488	0.333303	0.086165	0.082*	
C20	0.9824 (5)	0.4130 (9)	0.2065 (4)	0.0526 (14)	
H20	1.034536	0.532974	0.218520	0.063*	

### Atomic displacement parameters (Å^2^)

**Table:**

	*U*^11^	*U*^22^	*U*^33^	*U*^12^	*U*^13^	*U*^23^
Cl1	0.0550 (10)	0.0672 (12)	0.0641 (11)	−0.0116 (9)	0.0217 (8)	−0.0154 (10)
Cl1A	0.0550 (10)	0.0672 (12)	0.0641 (11)	−0.0116 (9)	0.0217 (8)	−0.0154 (10)
F1	0.134 (4)	0.130 (4)	0.094 (3)	−0.030 (3)	0.072 (3)	−0.054 (3)
O1	0.0436 (18)	0.038 (2)	0.074 (2)	0.0100 (16)	0.0311 (17)	−0.0024 (17)
O2	0.0387 (17)	0.052 (2)	0.063 (2)	−0.0123 (17)	0.0283 (16)	−0.0098 (18)
N1	0.035 (2)	0.034 (2)	0.054 (2)	0.0004 (17)	0.0227 (18)	−0.0041 (18)
N2	0.037 (2)	0.032 (2)	0.054 (2)	−0.0014 (17)	0.0195 (19)	−0.0036 (18)
N3	0.034 (2)	0.039 (2)	0.050 (2)	−0.0057 (18)	0.0216 (17)	−0.0019 (19)
C7	0.043 (6)	0.044 (4)	0.060 (5)	0.008 (5)	0.029 (6)	0.002 (5)
C1	0.026 (4)	0.049 (5)	0.050 (4)	0.007 (4)	0.017 (3)	−0.012 (4)
C2	0.037 (3)	0.043 (3)	0.051 (4)	−0.005 (2)	0.017 (3)	−0.021 (3)
C3	0.054 (5)	0.043 (5)	0.067 (6)	0.001 (4)	0.030 (5)	0.001 (4)
C4	0.055 (5)	0.060 (5)	0.074 (5)	0.007 (4)	0.036 (4)	0.009 (4)
C5	0.048 (5)	0.033 (4)	0.100 (8)	−0.008 (4)	0.041 (5)	−0.021 (5)
C6	0.037 (3)	0.043 (3)	0.051 (4)	−0.005 (2)	0.017 (3)	−0.021 (3)
C7A	0.043 (6)	0.044 (4)	0.060 (5)	0.008 (5)	0.029 (6)	0.002 (5)
C1A	0.026 (4)	0.049 (5)	0.050 (4)	0.007 (4)	0.017 (3)	−0.012 (4)
C2A	0.037 (3)	0.043 (3)	0.051 (4)	−0.005 (2)	0.017 (3)	−0.021 (3)
C3A	0.054 (5)	0.043 (5)	0.067 (6)	0.001 (4)	0.030 (5)	0.001 (4)
C4A	0.055 (5)	0.060 (5)	0.074 (5)	0.007 (4)	0.036 (4)	0.009 (4)
C5A	0.048 (5)	0.033 (4)	0.100 (8)	−0.008 (4)	0.041 (5)	−0.021 (5)
C6A	0.037 (3)	0.043 (3)	0.051 (4)	−0.005 (2)	0.017 (3)	−0.021 (3)
C8	0.035 (2)	0.037 (3)	0.045 (3)	0.001 (2)	0.016 (2)	−0.003 (2)
C9	0.036 (2)	0.037 (3)	0.051 (3)	0.003 (2)	0.022 (2)	−0.001 (2)
C10	0.035 (2)	0.033 (2)	0.046 (3)	0.002 (2)	0.017 (2)	−0.002 (2)
C11	0.033 (2)	0.035 (3)	0.048 (3)	0.000 (2)	0.015 (2)	−0.006 (2)
C12	0.050 (3)	0.043 (3)	0.067 (3)	0.006 (2)	0.028 (3)	−0.002 (3)
C13	0.037 (2)	0.041 (3)	0.059 (3)	−0.007 (2)	0.028 (2)	−0.009 (2)
C14	0.031 (2)	0.040 (3)	0.052 (3)	0.000 (2)	0.020 (2)	0.001 (2)
C15	0.033 (2)	0.048 (3)	0.045 (3)	0.000 (2)	0.017 (2)	0.001 (2)
C16	0.043 (3)	0.047 (3)	0.047 (3)	−0.003 (2)	0.018 (2)	−0.006 (3)
C17	0.058 (3)	0.058 (3)	0.059 (3)	−0.007 (3)	0.025 (3)	−0.013 (3)
C18	0.070 (4)	0.079 (4)	0.057 (3)	−0.007 (3)	0.031 (3)	−0.024 (3)
C19	0.068 (4)	0.096 (5)	0.057 (3)	−0.012 (4)	0.041 (3)	−0.010 (3)
C20	0.044 (3)	0.065 (4)	0.055 (3)	−0.003 (3)	0.026 (3)	0.003 (3)

### Geometric parameters (Å, º)

**Table:**

Cl1—C2	1.723 (4)	C1A—C6A	1.3900
Cl1A—C6A	1.666 (9)	C2A—C3A	1.3900
F1—C18	1.355 (7)	C2A—H2A	0.9500
O1—C10	1.237 (5)	C3A—C4A	1.3900
O2—C14	1.222 (5)	C3A—H3B	0.9500
N1—N2	1.362 (5)	C4A—C5A	1.3900
N1—C10	1.366 (6)	C4A—H4A	0.9500
N1—C13	1.450 (6)	C5A—C6A	1.3900
N2—C11	1.314 (6)	C5A—H5A	0.9500
N3—C14	1.356 (5)	C8—C9	1.340 (6)
N3—C15	1.410 (6)	C8—C11	1.435 (7)
N3—H3	0.8800	C9—C10	1.441 (6)
C7—C8	1.525 (6)	C9—H9	0.9500
C7—C1	1.529 (6)	C11—C12	1.490 (6)
C7—H7A	0.9900	C12—H12A	0.9800
C7—H7B	0.9900	C12—H12B	0.9800
C1—C2	1.3900	C12—H12C	0.9800
C1—C6	1.3900	C13—C14	1.512 (7)
C2—C3	1.3900	C13—H13A	0.9900
C3—C4	1.3900	C13—H13B	0.9900
C3—H3A	0.9500	C15—C16	1.376 (7)
C4—C5	1.3900	C15—C20	1.393 (6)
C4—H4	0.9500	C16—C17	1.384 (7)
C5—C6	1.3900	C16—H16	0.9500
C5—H5	0.9500	C17—C18	1.367 (8)
C6—H6	0.9500	C17—H17	0.9500
C7A—C8	1.524 (7)	C18—C19	1.360 (9)
C7A—C1A	1.529 (6)	C19—C20	1.378 (8)
C7A—H7C	0.9900	C19—H19	0.9500
C7A—H7D	0.9900	C20—H20	0.9500
C1A—C2A	1.3900		
			
N2—N1—C10	126.5 (4)	C6A—C5A—H5A	120.0
N2—N1—C13	113.1 (4)	C5A—C6A—C1A	120.0
C10—N1—C13	120.3 (4)	C5A—C6A—Cl1A	119.4 (7)
C11—N2—N1	117.3 (4)	C1A—C6A—Cl1A	120.5 (8)
C14—N3—C15	127.9 (4)	C9—C8—C11	118.6 (4)
C14—N3—H3	116.0	C9—C8—C7A	124.8 (6)
C15—N3—H3	116.0	C11—C8—C7A	115.8 (5)
C8—C7—C1	115.3 (4)	C9—C8—C7	123.1 (5)
C8—C7—H7A	108.4	C11—C8—C7	118.0 (4)
C1—C7—H7A	108.4	C8—C9—C10	121.4 (4)
C8—C7—H7B	108.4	C8—C9—H9	119.3
C1—C7—H7B	108.4	C10—C9—H9	119.3
H7A—C7—H7B	107.5	O1—C10—N1	121.0 (4)
C2—C1—C6	120.0	O1—C10—C9	124.9 (4)
C2—C1—C7	120.3 (7)	N1—C10—C9	114.1 (4)
C6—C1—C7	119.7 (7)	N2—C11—C8	122.0 (4)
C1—C2—C3	120.0	N2—C11—C12	114.9 (4)
C1—C2—Cl1	122.4 (3)	C8—C11—C12	123.1 (4)
C3—C2—Cl1	117.6 (3)	C11—C12—H12A	109.5
C4—C3—C2	120.0	C11—C12—H12B	109.5
C4—C3—H3A	120.0	H12A—C12—H12B	109.5
C2—C3—H3A	120.0	C11—C12—H12C	109.5
C3—C4—C5	120.0	H12A—C12—H12C	109.5
C3—C4—H4	120.0	H12B—C12—H12C	109.5
C5—C4—H4	120.0	N1—C13—C14	112.8 (4)
C6—C5—C4	120.0	N1—C13—H13A	109.0
C6—C5—H5	120.0	C14—C13—H13A	109.0
C4—C5—H5	120.0	N1—C13—H13B	109.0
C5—C6—C1	120.0	C14—C13—H13B	109.0
C5—C6—H6	120.0	H13A—C13—H13B	107.8
C1—C6—H6	120.0	O2—C14—N3	125.2 (4)
C8—C7A—C1A	112.7 (6)	O2—C14—C13	122.9 (4)
C8—C7A—H7C	109.1	N3—C14—C13	111.9 (4)
C1A—C7A—H7C	109.1	C16—C15—C20	119.4 (4)
C8—C7A—H7D	109.1	C16—C15—N3	124.1 (4)
C1A—C7A—H7D	109.1	C20—C15—N3	116.5 (4)
H7C—C7A—H7D	107.8	C15—C16—C17	120.4 (5)
C2A—C1A—C6A	120.0	C15—C16—H16	119.8
C2A—C1A—C7A	118.8 (17)	C17—C16—H16	119.8
C6A—C1A—C7A	121.2 (17)	C18—C17—C16	118.4 (5)
C1A—C2A—C3A	120.0	C18—C17—H17	120.8
C1A—C2A—H2A	120.0	C16—C17—H17	120.8
C3A—C2A—H2A	120.0	F1—C18—C19	117.9 (5)
C4A—C3A—C2A	120.0	F1—C18—C17	119.1 (5)
C4A—C3A—H3B	120.0	C19—C18—C17	123.0 (5)
C2A—C3A—H3B	120.0	C18—C19—C20	118.4 (5)
C5A—C4A—C3A	120.0	C18—C19—H19	120.8
C5A—C4A—H4A	120.0	C20—C19—H19	120.8
C3A—C4A—H4A	120.0	C19—C20—C15	120.4 (5)
C4A—C5A—C6A	120.0	C19—C20—H20	119.8
C4A—C5A—H5A	120.0	C15—C20—H20	119.8
			
C10—N1—N2—C11	−1.5 (6)	C7—C8—C9—C10	174.7 (9)
C13—N1—N2—C11	179.6 (4)	N2—N1—C10—O1	−178.3 (4)
C8—C7—C1—C2	98.6 (11)	C13—N1—C10—O1	0.6 (6)
C8—C7—C1—C6	−80.7 (11)	N2—N1—C10—C9	2.5 (6)
C6—C1—C2—C3	0.0	C13—N1—C10—C9	−178.7 (4)
C7—C1—C2—C3	−179.3 (3)	C8—C9—C10—O1	179.2 (5)
C6—C1—C2—Cl1	−179.3 (4)	C8—C9—C10—N1	−1.6 (6)
C7—C1—C2—Cl1	1.4 (4)	N1—N2—C11—C8	−0.5 (6)
C1—C2—C3—C4	0.0	N1—N2—C11—C12	179.5 (4)
Cl1—C2—C3—C4	179.3 (4)	C9—C8—C11—N2	1.2 (7)
C2—C3—C4—C5	0.0	C7A—C8—C11—N2	171.4 (18)
C3—C4—C5—C6	0.0	C7—C8—C11—N2	−173.8 (8)
C4—C5—C6—C1	0.0	C9—C8—C11—C12	−178.7 (4)
C2—C1—C6—C5	0.0	C7A—C8—C11—C12	−8.6 (18)
C7—C1—C6—C5	179.3 (3)	C7—C8—C11—C12	6.2 (10)
C8—C7A—C1A—C2A	107 (2)	N2—N1—C13—C14	−105.4 (4)
C8—C7A—C1A—C6A	−73 (2)	C10—N1—C13—C14	75.6 (5)
C6A—C1A—C2A—C3A	0.0	C15—N3—C14—O2	−7.4 (7)
C7A—C1A—C2A—C3A	179.9 (4)	C15—N3—C14—C13	172.5 (4)
C1A—C2A—C3A—C4A	0.0	N1—C13—C14—O2	−7.6 (7)
C2A—C3A—C4A—C5A	0.0	N1—C13—C14—N3	172.5 (4)
C3A—C4A—C5A—C6A	0.0	C14—N3—C15—C16	−8.1 (7)
C4A—C5A—C6A—C1A	0.0	C14—N3—C15—C20	174.3 (4)
C4A—C5A—C6A—Cl1A	−178.2 (7)	C20—C15—C16—C17	1.9 (7)
C2A—C1A—C6A—C5A	0.0	N3—C15—C16—C17	−175.5 (5)
C7A—C1A—C6A—C5A	−179.9 (4)	C15—C16—C17—C18	−1.1 (8)
C2A—C1A—C6A—Cl1A	178.2 (7)	C16—C17—C18—F1	−179.4 (5)
C7A—C1A—C6A—Cl1A	−1.8 (8)	C16—C17—C18—C19	−0.8 (9)
C1A—C7A—C8—C9	−13 (3)	F1—C18—C19—C20	−179.6 (6)
C1A—C7A—C8—C11	177.5 (16)	C17—C18—C19—C20	1.8 (10)
C1—C7—C8—C9	−5.9 (15)	C18—C19—C20—C15	−1.0 (9)
C1—C7—C8—C11	168.9 (8)	C16—C15—C20—C19	−0.9 (8)
C11—C8—C9—C10	−0.1 (7)	N3—C15—C20—C19	176.8 (5)
C7A—C8—C9—C10	−169.2 (19)		

### Hydrogen-bond geometry (Å, º)

**Table:**

*D*—H···*A*	*D*—H	H···*A*	*D*···*A*	*D*—H···*A*
N3—H3···O1^i^	0.88	1.95	2.815 (5)	168
C7—H7*A*···O2^ii^	0.99	2.32	3.282 (15)	164
C16—H16···O2	0.95	2.29	2.900 (6)	121
C19—H19···F1^iii^	0.95	2.42	3.336 (7)	162

Symmetry codes: (i) −*x*+2, *y*+1/2, −*z*+1; (ii) −*x*+1, *y*+1/2, −*z*+1; (iii) −*x*+2, *y*+1/2, −*z*.

## References

[bb1] Abiha, G. B., Bahar, L. & Utku, S. (2018). *Rev. Rom. Med. Lab.* **26**, 231–241.

[bb2] Boukharsa, Y., Meddah, B., Tiendrebeogo, R. Y., Ibrahimi, A., Taoufik, J., Cherrah, Y., Benomar, A., Faouzi, M. E. A. & Ansar, M. (2016). *Med. Chem. Res.* **25**, 494–500.

[bb3] Brandenburg, K. & Putz, H. (2012). *DIAMOND*, Crystal Impact GbR, Bonn, Germany.

[bb4] Bruker (2021). *APEX4 and *SAINT* * . Bruker AXS LLC, Madison, Wisconsin, USA.

[bb5] Ibrahim, T. H., Loksha, Y. M., Elshihawy, H. A., Khodeer, D. M. & Said, M. M. (2017). *Arch. Pharm. Chem. Life Sci.* **350**, e1700093. 10.1002/ardp.201700093 28792072

[bb6] Kamble, V. T., Sawant, A.-S., Sawant, S. S., Pisal, P. M., Gacche, R. N., Kamble, S. S., Shegokar, H. D. & Kamble, V. A. (2017). *J. Basic Appl. Res. Int.* **21**, 10–39.

[bb7] Khokra, S. L., Khan, S. A., Thakur, P., Chowdhary, D., Ahmad, A. & Husain, A. (2016). *J. Chin. Chem. Soc.* **63**, 739–750.

[bb8] Krause, L., Herbst-Irmer, R., Sheldrick, G. M. & Stalke, D. (2015). *J. Appl. Cryst.* **48**, 3–10. 10.1107/S1600576714022985 PMC4453166 26089746

[bb9] Missioui, M., Said, M. A., Demirtaş, G., Mague, J. T. & Ramli, Y. (2022). *J. Mol. Struct.* **1247**, 131420.

[bb10] Mortada, S., Guerrab, W., Missioui, M., Salhi, N., Naceiri Mrabti, H., Rouass, L., Benkirane, S., Hassane, M., Masrar, A., Mezzour, H., Faouzi, M. E. A. & Ramli, Y. (2023). *J. Biomol. Struct. Dyn.* pp. 1–15. 10.1080/07391102.2023.2246574 37583282

[bb11] Parsons, S., Flack, H. D. & Wagner, T. (2013). *Acta Cryst.* B**69**, 249–259. 10.1107/S2052519213010014 PMC3661305 23719469

[bb12] Sheldrick, G. M. (2008). *Acta Cryst.* A**64**, 112–122. 10.1107/S0108767307043930 18156677

[bb13] Sheldrick, G. M. (2015*a*). *Acta Cryst.* A**71**, 3–8.

[bb14] Sheldrick, G. M. (2015*b*). *Acta Cryst.* C**71**, 3–8.

[bb15] Yamada, T., Nobuhara, Y., Shimamura, H., Yoshihara, K., Yamaguchi, A. & Ohki, M. (1981). *Chem. Pharm. Bull.* **29**, 3433–3439. 10.1248/cpb.29.3433 7340942

[bb16] Zaoui, Y., Assila, H., Mague, J. T., Alsubari, A., Taoufik, J., Ramli, Y. & Ansar, M. (2022). *IUCrData*, **7**, x220582. 10.1107/S241431462200582X PMC9462039 36339895

